# Physical-Layer Security Analysis over M-Distributed Fading Channels

**DOI:** 10.3390/e21100998

**Published:** 2019-10-12

**Authors:** Sheng-Hong Lin, Rong-Rong Lu, Xian-Tao Fu, An-Ling Tong, Jin-Yuan Wang

**Affiliations:** 1 Key Laboratory of Intelligent Computing & Signal Processing, Ministry of Education, Anhui University, Hefei 230039, China; 2 Shanghai Key Laboratory of Integrated Administration Technologies for Information Security, Shanghai Jiao Tong University, Shanghai 200240, China; 3 Key Lab of Broadband Wireless Communication and Sensor Network Technology, Ministry of Education, Nanjing University of Posts and Telecommunications, Nanjing 210003, China; 4 General Information Department, North Information Control Research Academy Group Co., Ltd., Nanjing 21153, China

**Keywords:** physical layer security, M-distributed fading channels, SOP, SPSC, ESC

## Abstract

In this paper, the physical layer security over the M-distributed fading channel is investigated. Initially, an exact expression of secrecy outage probability (SOP) is derived, which has an integral term. To get a closed-form expression, a lower bound of SOP is obtained. After that, the exact expression for the probability of strictly positive secrecy capacity (SPSC) is derived, which is in closed-form. Finally, an exact expression of ergodic secrecy capacity (ESC) is derived, which has two integral terms. To reduce its computational complexity, a closed-from expression for the lower bound of ESC is obtained. As special cases of M-distributed fading channels, the secure performance of the K, exponential, and Gamma-Gamma fading channels are also derived, respectively. Numerical results show that all theoretical results match well with Monte-Carlo simulation results. Specifically, when the average signal-to-noise ratio of main channel is larger than 40 dB, the relative errors for the lower bound of SOP, the probability of SPSC, and the lower bound of ESC are less than 1.936%, 6.753%, and 1.845%, respectively. This indicates that the derived theoretical expressions can be directly used to evaluate system performance without time-consuming simulations. Moreover, the derived results regarding parameters that influence the secrecy performance will enable system designers to quickly determine the optimal available parameter choices when facing different security risks.

## 1. Introduction

For future wireless communications, it can be expected that it will be necessary to support massive user connections and exponentially increasing wireless services [1]. With the explosive growth of wireless services, information privacy and security are becoming a major growing concern for users [2]. Traditionally, classical network security techniques [3] are often employed. However, these techniques do not consider the physical nature of the wireless channel. Recently, as a compelling technology for supplementing traditional network security, physical layer security (PLS) has been proposed [4].

The framework of PLS was pioneered by Shannon, who proposed the concept of perfect secrecy over noiseless channels [5]. However, random noise is an intrinsic element of almost all wireless communication channels. Under noisy channels, Wyner established the wiretap channel model and proposed the concept of secrecy capacity [5]. For Wyner’s wiretap model, a passive eavesdropper wiretapped the transmission over the main channel. While attempting to decode the information, no conditions are imposed on the available resources for the eavesdropper. After that, Csiszár introduced the non-degraded channels [5]. It was proved that the secure communication over additive white Gaussian noise (AWGN) channels can be achieved as long as the channel capacity of the main channel is better than that of the eavesdropping channel.

By considering the impacts of channel fadings in wireless communications, recent works extended previous results to many scenarios. Under complex Gaussian fading channels, the secrecy capacity was derived in [6]. For Rayleigh fading channels, the average secrecy capacity and secrecy outage performance for frequency diverse array communications were investigated in [7]. In [8], the average secrecy outage probability (SOP) and the average secrecy outage duration of Nakagami-m fading channel were discussed. Under generalized Gamma fading channels, the theoretical expressions of the probability of strictly positive secrecy capacity (SPSC) and the SOP were derived in [9]. Moreover, for Nakagami-n (i.e., Rice) fading channel, the probability of SPSC was derived [10]. Another commonly used model is called lognormal fading, which was usually employed to characterize the shadow fading in radio frequency wireless communications (RFWC) [11], the atmosphere turbulence effects in optical wireless communications (OWC) [12], or the small-scale fading for indoor ultra wide band (UWB) communications [13]. In [14], the SOP of the correlated lognormal fading channels was discussed. For independent/correlated lognormal fading channels, the ergodic secrecy capacity (ESC) and the SOP were investigated in [15]. Moreover, in [16] and [17], the secrecy capacities were analyzed over generalized-K fading channel and κ−μ fading channel, respectively. As mentioned above, the PLS for many well-known fading channels have been discussed. However, to the best of our knowledge, the PLS for a newly proposed fading model, M distribution, has not been involved in open literature.

One motivation to consider the M distribution in this paper is its generality. The M distribution is a general fading model since it covers many well-known fading models as special cases, such as lognormal, Gamma, K, Gamma-Gamma, exponential, et al. [18]. In other words, the M-distributed fading model unifies many previously proposed fading models in a single one. Another motivation is that the M-distributed fading can be applied to many communication environments. Specifically, it can be used to characterize the shadow fading in RFWC, the atmosphere turbulence effects in OWC, or the small-scale fading in indoor UWB communications. As a new tractable fading model, it has been widely used to describe the statistical behavior of these wireless channels. Up to now, closed-form expressions of different performance indicators (such as bit error rate [19], outage probability [20] and capacity [21]) over M fading channels had been analyzed. However, the secure performance over M fading channels has not been completely revealed.

In this paper, a wireless communication network with one transmitter (Alice), one legitimate receiver (Bob) and one eavesdropper (Eve) is considered. The main and eavesdropping channels experience independent M-distributed fadings. The PLS over the M fading channel is investigated. The main contributions are listed as follows:

- Under the M-distributed fading channel, the exact expression of the SOP is first derived. To reduce computational complexity, a closed-form expression for the lower bound of the SOP is obtained. It is shown that the performance gap between the exact SOP expression and its lower bound is small in the high signal-to-noise ratio (SNR) regime.
- The closed-form expression for the probability of the SPSC over the M-distributed fading channel is derived. It is found that when the target secrecy rate is set to be zero, the lower bound of SOP has the same expression as the probability of the SPSC.
- Over the M-distributed fading channel, the exact expression of the ESC with a double integral is obtained. To obtain a closed-form expression, the lower bound of ESC is then derived. It is shown that the performance gap between the exact expression and the lower bound of the ESC is small.
- As special cases of the M fading channel, the secure performance over the K, exponential, and Gamma-Gamma fading channels is derived, respectively. The accuracy of these performance analysis is verified by simulations.

The remainder of this paper is organized as follows. In Section 2, the system model is introduced. Section 3 focuses on the secure performance analysis. For some special cases, the secrecy performance is further investigated in Section 4. Section 5 presents numerical results before Section 6 concludes the paper.

*Notations*: E(·) denotes the expectation operator, Var(·) denotes the variance operator. Γ(·) denotes the Gamma function. $K_{v}\left(\cdot \right)$ denotes the modified Bessel function of the second kind with order *v*. $G_{p,q}^{m,n}\left[\cdot |\cdot \right]$ denotes the Meijer’s G function [22]. N(a,b) denotes a Gaussian distribution with mean *a* and variance *b*. $f_{X}\left(x\right)$ and $F_{X}\left(x\right)$ denote the probability density function (PDF) and the cumulative distribution function (CDF) of a random variance *X*. ${\left\{x\right\}}^{+}$ denotes max{x,0}.

## 2. System Model

**Figure 1:**
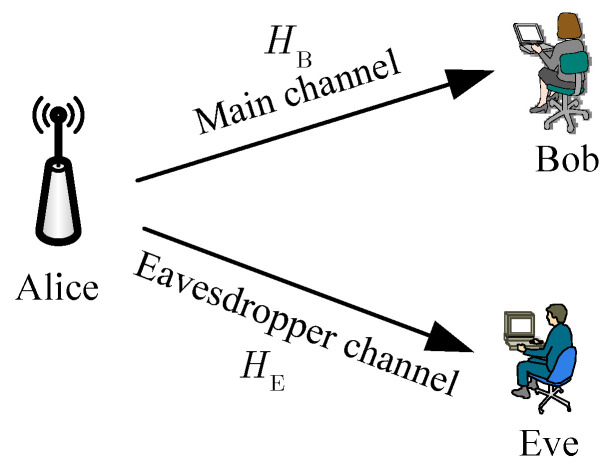
A wireless communication system with a transmitter (Alice), a legitimate receiver (Bob), and an eavesdropper (Eve).

In this paper, we consider a wireless communication system with three entities, i.e., a transmitter (Alice), a legitimate receiver (Bob), and an eavesdropper (Eve), as depicted in Figure 1. In the system, Alice transmits data to Bob. Due to the broadcast feature of the wireless channel, both Bob and Eve can receive the signal.

For Bob and Eve, the received signals $Y_{n}$ (n=B for Alice-Bob link and n=E for Alice-Eve link) are given by

(1) $$\begin{array}{c} Y_{n}=H_{n}X+Z_{n} \end{array}$$

where *X* is the transmit signal, and it satisfies $E(X^{2})=P$, where *P* is the average power of the transmit signal. $Z_{n}∼N\left(0,\sigma_{n}^{2}\right)$ stands for the AWGN, where $\sigma_{n}^{2}$ is the noise variance. $H_{n}$ denotes the M-distributed channel fading, which is a seven-parameter distribution and can be denoted as $H_{n}∼M\left(\alpha_{n},\beta_{n},\rho_{n},\Omega_{n},\xi_{n},\phi_{1,n},\phi_{2,n}\right)$. The PDF of $H_{n}$ is given by [19]

(2) $$f_{H_{n}}\left(h\right)=A_{n}\sum_{k=1}^{\beta_{n}}a_{k,n}h_{n}^{\frac{\alpha_{n}+k}{2}-1}K_{\alpha_{n}-k}\left(2\sqrt{\frac{\alpha_{n}\beta_{n}h}{\xi_{g,n}\beta_{n}+\Omega_{n}^{\prime }}}\right),\;h\geq 0$$

where

(3) $$\left\{\begin{array}{c} A_{n}=\frac{2\alpha_{n}^{\frac{\alpha_{n}}{2}}}{\xi_{g,n}^{1+\frac{\alpha_{n}}{2}}\Gamma \left(\alpha_{n}\right)}{\left(\frac{\xi_{g,n}\beta_{n}}{\xi_{g,n}\beta_{n}+\Omega_{n}^{\prime }}\right)}^{\beta_{n}+\frac{\alpha_{n}}{2}}\\ a_{k,n}=\left(\begin{array}{c} \beta_{n}-1\\ k-1 \end{array}\right)\frac{{\left(\xi_{g,n}\beta +\Omega_{n}^{\prime }\right)}^{1-\frac{k}{2}}}{\Gamma (k)}{\left(\frac{\Omega_{n}^{\prime }}{\xi_{g,n}}\right)}^{k-1}{\left(\frac{\alpha_{n}}{\beta_{n}}\right)}^{\frac{k}{2}}\\ \Omega_{n}^{\prime }=\Omega_{n}+\xi_{c,n}+2\sqrt{\xi_{c,n}\Omega_{n}}\cos\left(\phi_{1,n}-\phi_{2,n}\right)\\ \xi_{c,n}=\rho_{n}\xi_{n}\\ \xi_{g,n}=\left(1-\rho_{n}\right)\xi_{n} \end{array}\right.$$

Moreover, $\alpha_{n}>0$ is a parameter related to the effective number of large-scale cells of the scattering process. $\beta_{n}\in N$ denotes the amount of fading parameter. $\Omega_{n}^{\prime }$ denotes the average power of the LOS component and the coupled-to-LOS scattering term. $\phi_{1,n}$ and $\phi_{2,n}$ are the deterministic phases of the LOS and the coupled-to-LOS scatter components, respectively. $\xi_{n}$ is the average power of the total scatter components, and $\rho_{n}$ is a parameter in the range of [0,1].

From (1), the received SNRs at Bob and Eve are written as

(4) γn=PHn2PHn2σn2σn2

According to (2) and (4), the PDF of $\gamma_{n}$ is given by

(5) $$\begin{array}{c} f_{\gamma_{n}}\left(r\right)=\frac{A_{n}}{2}\sum_{j=1}^{\beta_{n}}a_{j,n}{\left(\frac{\sigma_{n}}{\sqrt{P}}\right)}^{\frac{\alpha_{n}+j}{2}}r^{\frac{\alpha_{n}+j}{4}-1}K_{\alpha_{n}-j}\left(2\sqrt{\frac{\alpha_{n}\beta_{n}\sigma_{n}\sqrt{r}}{\sqrt{P}\left(\xi_{g,n}\beta_{n}+\Omega_{n}^{\prime }\right)}}\right),r\geq 0 \end{array}$$

Moreover, the CDF of $\gamma_{n}$ can be derived as

(6) $$\begin{array}{c} F_{\gamma_{n}}\left(r\right)=A_{n}\sum_{k=1}^{\beta_{n}}a_{k,n}{\left(\frac{\sigma_{n}}{\sqrt{P}}\right)}^{\frac{\alpha_{n}+k}{2}}\int_{0}^{\sqrt{r}}m^{\frac{\alpha_{n}+k}{2}-1}K_{\alpha_{n}-k}\left(2\sqrt{\frac{\alpha_{n}\beta_{n}\sigma_{n}m}{\sqrt{P}\left(\xi_{g,n}\beta_{n}+\Omega_{n}^{\prime }\right)}}\right)dm \end{array}$$

According to (14) in [20], $K_{v}\left(x\right)$ in (6) can be rewritten as Meijer’s G-function, and then using (26) in [20] for (6), the CDF of $\gamma_{n}$ is given by

(7) $$\begin{array}{c} F_{\gamma_{n}}\left(r\right)=\frac{A_{n}}{2}\sum_{j=1}^{\beta_{n}}a_{j,n}{\left(\frac{\sigma_{n}}{\sqrt{P}}\right)}^{\frac{\alpha_{n}+j}{2}}r^{\frac{\alpha_{n}+j}{4}}G_{1,3}^{2,1}\left[\frac{\alpha_{n}\beta_{n}\sigma_{n}\sqrt{r}}{\sqrt{P}\left(\xi_{g,n}\beta_{n}+\Omega_{n}^{\prime }\right)}\left|\begin{array}{c} 1-\frac{\alpha_{n}+j}{2}\\ \frac{\alpha_{n}-j}{2},\frac{j-\alpha_{n}}{2},-\frac{\alpha_{n}+j}{2} \end{array}\right.\right] \end{array}$$

## 3. Secrecy Performance Analysis

In the following three subsections, the SOP, the probability of SPSC, and the ESC over the M-distributed fading channel will be discussed, respectively.

### 3.1. SOP Analysis

According to [23], the secrecy capacity for one realization of the SNR pair $\left(\gamma_{B},\gamma_{E}\right)$ is given by

(8) $$C=\left\{\begin{array}{cc} \ln\left(1+\gamma_{B}\right)-\ln\left(1+\gamma_{E}\right), & \;\mathrm{if}\;\gamma_{B}>\gamma_{E}\\ 0, & \;\mathrm{if}\;\gamma_{B}\leq \gamma_{E} \end{array}\right.$$

Referring to (8), the SOP is defined as the probability that the instantaneous secrecy capacity falls below a target secrecy rate, which can be written as

(9) $$P_{\mathrm{SOP}}=\mathrm{Pr}\left(C\leq c\right)$$

where *c* denotes the target secrecy rate, and it can be re-expressed using the SNR threshold $\gamma_{\mathrm{th}}$ as $c=\ln(1+\gamma_{\mathrm{th}})$. Therefore, Equation (9) can be further written as

(10) $$\begin{array}{ccc} P_{\mathrm{SOP}} & = & \mathrm{Pr}\left[\ln\left(\frac{1+\gamma_{B}}{1+\gamma_{E}}\right)\leq \ln\left(1+\gamma_{\mathrm{th}}\right)\right]\\ & = & \mathrm{Pr}\left[\gamma_{B}\leq \left(1+\gamma_{\mathrm{th}}\right)\left(1+\gamma_{E}\right)-1\right] \end{array}$$

According to (5), Equation (10) can be further written as

(11) $$\begin{array}{c} P_{\mathrm{SOP}}=\int_{0}^{\infty }\int_{0}^{\left(1+\gamma_{\mathrm{th}}\right)\left(1+w\right)-1}f_{\gamma_{E}}\left(w\right)f_{\gamma_{B}}\left(u\right)dudw \end{array}$$

By analyzing (11), the double integrals can be simplified to a single one, which is shown in the following theorem.

**Theorem** **1.** *For the M-distributed fading channels (i.e., $H_{n}∼M\left(\alpha_{n},\beta_{n},\rho_{n},\Omega_{n},\xi_{n},\phi_{1,n},\phi_{2,n}\right)$, the SOP can be derived as a single integral*

(12) $$\begin{array}{ccc} P_{\mathrm{SOP}} & = & 1-\frac{\left(1+\gamma_{\mathrm{th}}\right)A_{B}A_{E}}{4}\sum_{k=1}^{\beta_{E}}\sum_{j=1}^{\beta_{B}}a_{k,E}{\left(\frac{\sigma_{E}}{\sqrt{P}}\right)}^{\frac{\alpha_{E}+k}{2}}a_{j,B}{\left(\frac{\sigma_{B}}{\sqrt{P}}\right)}^{\frac{\alpha_{B}+j}{2}}\int_{0}^{\infty }w^{\frac{\alpha_{E}+k}{4}}{\left[\left(1+\gamma_{\mathrm{th}}\right)\left(1+w\right)-1\right]}^{\frac{\alpha_{B}+j}{4}-1}\\ & \times & G_{1,3}^{2,1}\left[\frac{\alpha_{E}\beta_{E}\sigma_{E}\sqrt{w}}{\sqrt{P}\left(\xi_{g,E}\beta_{E}+\Omega_{E}^{\prime }\right)}\left|\begin{array}{c} 1-\frac{\alpha_{E}+k}{2}\\ \frac{\alpha_{E}-k}{2},-\frac{\alpha_{E}-k}{2},-\frac{\alpha_{E}+k}{2} \end{array}\right.\right]K_{\alpha_{B}-j}\left(2\sqrt{\frac{\alpha_{B}\beta_{B}\sigma_{B}\sqrt{\left(1+\gamma_{\mathrm{th}}\right)\left(1+w\right)-1}}{\sqrt{P}\left(\xi_{g,B}\beta_{B}+\Omega_{B}^{\prime }\right)}}\right)dw \end{array}$$

**Proof.**  According to (11) and using the partial integration, the SOP can be further written as

(13) $$\begin{array}{ccc} P_{\mathrm{SOP}} & = & \int_{0}^{\infty }F_{\gamma_{B}}\left[\left(1+\gamma_{\mathrm{th}}\right)\left(1+w\right)-1\right]dF_{\gamma_{E}}\left(w\right)\\ & = & 1-\left(1+\gamma_{\mathrm{th}}\right)\int_{0}^{\infty }F_{\gamma_{E}}\left(w\right)f_{\gamma_{B}}\left[\left(1+\gamma_{\mathrm{th}}\right)\left(1+w\right)-1\right]dw \end{array}$$

Substituting (5) and (7) into (13), Equation (12) can be obtained. □

**Remark** **1.** *The SOP $P_{\mathrm{SOP}}$ is a monotonously increasing function with respect to the SNR threshold $\gamma_{\mathrm{th}}$. Therefore, the minimum and maximum values of the SOP are given by $P_{\mathrm{SOP}}^{\min}=P_{\mathrm{SOP}}\left(\gamma_{\mathrm{th}}=0\right)$ and $P_{\mathrm{SOP}}^{\max}=P_{\mathrm{SOP}}\left(\gamma_{\mathrm{th}}\to \infty \right)=1$, respectively.*

**Figure 2:**
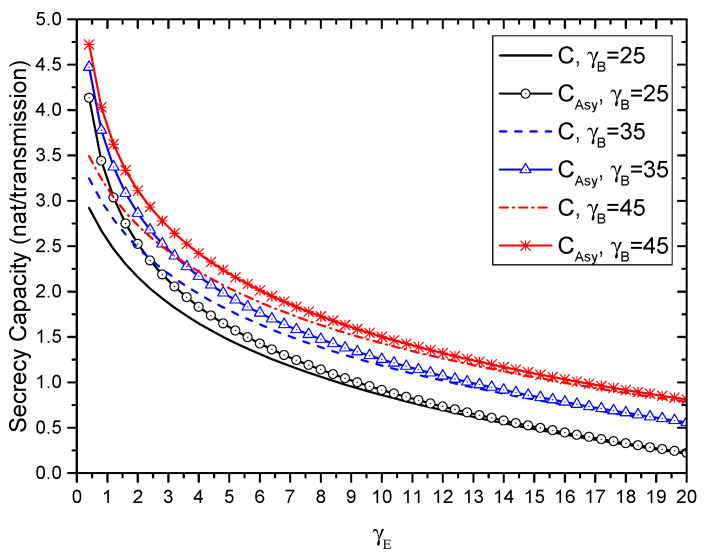
The instantaneous secrecy capacity *C* and the asymptotic secrecy capacity $C_{\mathrm{Asy}}$ for different $\gamma_{B}$.

Theoretically, Equation (12) can be employed to evaluate the SOP over the M-distributed fading channels. However, the expression has a complex integral term. Generally, we are more interested in the SOP performance at high SNR (i.e., $\gamma_{B}\gg 1,\gamma_{E}\gg 1$). In this case, a tight lower bound of the SOP will be provided in this paper. When $\gamma_{B}>\gamma_{E}$, we have $\left(1+\gamma_{B}\right)/\left(1+\gamma_{E}\right)<\gamma_{B}/\gamma_{E}$. Therefore, we have

(14) $$C=\ln\left(\frac{1+\gamma_{B}}{1+\gamma_{E}}\right)<\ln\left(\frac{\gamma_{B}}{\gamma_{E}}\right)\overset{\Delta }{\;=}C_{\mathrm{Asy}}$$

where $C_{\mathrm{Asy}}=C+\delta ,\;\left(\delta >0\right)$ can be interpreted as the asymptotic secrecy capacity. Figure 2 shows the instantaneous secrecy capacity *C* and the asymptotic secrecy capacity $C_{\mathrm{Asy}}$ for different $\gamma_{B}$. As can be seen, when $\gamma_{B}>\gamma_{E}\gg 1$, the performance gap between *C* and $C_{\mathrm{Asy}}$ is small. In other words, $C_{\mathrm{Asy}}$ can be used to evaluate SOP in the high SNR regime.

According to (14), the SOP can be written as

(15) $$\begin{array}{ccc} P_{\mathrm{SOP}} & = & \mathrm{Pr}\left[C\leq \ln(1+\gamma_{\mathrm{th}})\right]\\ & = & \mathrm{Pr}\left(C_{\mathrm{Asy}}-\delta \leq \ln\left(1+\gamma_{\mathrm{th}}\right)\right)\\ & \geq & \mathrm{Pr}\left(C_{\mathrm{Asy}}\leq \ln\left(1+\gamma_{\mathrm{th}}\right)\right)\\ & = & \mathrm{Pr}\left[\gamma_{B}\leq \left(1+\gamma_{\mathrm{th}}\right)\gamma_{E}\right]\overset{\Delta }{\;=}P_{\mathrm{SOP}}^{L} \end{array}$$

Note that (15) provides a lower bound $P_{\mathrm{SOP}}^{L}$ for the SOP. After some analysis, the following theorem is derived.

**Theorem** **2.** *For the M-distributed fading channels (i.e., $H_{n}∼M\left(\alpha_{n},\beta_{n},\rho_{n},\Omega_{n},\xi_{n},\phi_{1,n},\phi_{2,n}\right)$, a closed-form expression for the lower bound of SOP can be derived as*

(16) $$\begin{array}{ccc} P_{\mathrm{SOP}}^{L} & = & 1-\frac{A_{B}A_{E}}{4}\sum_{k=1}^{\beta_{E}}\sum_{j=1}^{\beta_{B}}a_{k,E}a_{j,B}{\left(\frac{\sigma_{B}\sqrt{1+\gamma_{\mathrm{th}}}}{\sigma_{E}}\right)}^{\frac{\alpha_{B}+j}{2}}{\left(\frac{\alpha_{E}\beta_{E}}{\xi_{g,E}\beta_{E}+\Omega_{E}^{\prime }}\right)}^{-\frac{\alpha_{E}+\alpha_{B}+k+j}{2}}\\ & \times & G_{3,3}^{3,2}\left[\chi \left(\gamma_{\mathrm{th}}\right)\left|\begin{array}{c} 1-\frac{\alpha_{B}+j}{2}-\alpha_{E},1-\frac{\alpha_{B}+j}{2}-k,1-\frac{\alpha_{B}+j}{2}\\ \frac{\alpha_{B}-j}{2},-\frac{\alpha_{B}-j}{2},-\frac{\alpha_{B}+j}{2} \end{array}\right.\right] \end{array}$$

*where*

(17) $$\begin{array}{c} \chi \left(\gamma_{\mathrm{th}}\right)\overset{\Delta }{\;=}\frac{\alpha_{B}\beta_{B}\sigma_{B}\left(\xi_{g,E}\beta_{E}+\Omega_{E}^{\prime }\right)\sqrt{1+\gamma_{\mathrm{th}}}}{\alpha_{E}\beta_{E}\sigma_{E}\left(\xi_{g,B}\beta_{B}+\Omega_{B}^{\prime }\right)} \end{array}$$

**Proof.**  By comparing (10) with (15), the lower bound of the SOP can be derived by replacing $\left(1+\gamma_{\mathrm{th}}\right)\left(1+w\right)-1$ with $(1\;+\;\gamma_{\mathrm{th}})w$ in (12), i.e.,

(18) $$\begin{array}{ccc} P_{\mathrm{SOP}}^{L} & = & 1-\frac{\left(1+\gamma_{\mathrm{th}}\right)A_{B}A_{E}}{4}\sum_{k=1}^{\beta_{E}}\sum_{j=1}^{\beta_{B}}a_{k,E}{\left(\frac{\sigma_{E}}{\sqrt{P}}\right)}^{\frac{\alpha_{E}+k}{2}}a_{j,B}{\left(\frac{\sigma_{B}}{\sqrt{P}}\right)}^{\frac{\alpha_{B}+j}{2}}\int_{0}^{\infty }w^{\frac{\alpha_{E}+k}{4}}{\left[(1+\gamma_{\mathrm{th}})w\right]}^{\frac{\alpha_{B}+j}{4}-1}\\ & \times & G_{1,3}^{2,1}\left[\frac{\alpha_{E}\beta_{E}\sigma_{E}\sqrt{w}}{\sqrt{P}\left(\xi_{g,E}\beta_{E}+\Omega_{E}^{\prime }\right)}\left|\begin{array}{c} 1-\frac{\alpha_{E}+k}{2}\\ \frac{\alpha_{E}-k}{2},-\frac{\alpha_{E}-k}{2},-\frac{\alpha_{E}+k}{2} \end{array}\right.\right]K_{\alpha_{B}-j}\left(2\sqrt{\frac{\alpha_{B}\beta_{B}\sigma_{B}\sqrt{(1+\gamma_{\mathrm{th}})w}}{\sqrt{P}\left(\xi_{g,B}\beta_{B}+\Omega_{B}^{\prime }\right)}}\right)dw \end{array}$$

By letting $t=\sqrt{w}$ and then using (14) in [20], Equation (18) can be written as

(19) $$\begin{array}{ccc} P_{\mathrm{SOP}}^{L} & = & 1-\frac{A_{B}A_{E}}{4}\sum_{k=1}^{\beta_{E}}\sum_{j=1}^{\beta_{B}}a_{k,E}{\left(\frac{\sigma_{E}}{\sqrt{P}}\right)}^{\frac{\alpha_{E}+k}{2}}a_{j,B}{\left(\frac{\sigma_{B}}{\sqrt{P}}\right)}^{\frac{\alpha_{B}+j}{2}}{\left(1+\gamma_{\mathrm{th}}\right)}^{\frac{\alpha_{B}+j}{4}}\int_{0}^{\infty }t^{\frac{\alpha_{E}+k}{2}+\frac{\alpha_{B}+j}{2}-1}\\ & \times & G_{1.3}^{2,1}\left[\frac{\alpha_{E}\beta_{E}\sigma_{E}t}{\sqrt{P}\left(\xi_{g,E}\beta_{E}+\Omega_{E}^{\prime }\right)}\left|\begin{array}{c} 1-\frac{\alpha_{E}+k}{2}\\ \frac{\alpha_{E}-k}{2},-\frac{\alpha_{E}-k}{2},-\frac{\alpha_{E}+k}{2} \end{array}\right.\right]G_{0,2}^{2,0}\left[\frac{\alpha_{B}\beta_{B}\sigma_{B}\sqrt{1+\gamma_{\mathrm{th}}}t}{\sqrt{P}\left(\xi_{g,B}\beta_{B}+\Omega_{B}^{\prime }\right)}\left|\begin{array}{c} -\\ \frac{\alpha_{B}-j}{2},-\frac{\alpha_{B}-j}{2} \end{array}\right.\right]dt \end{array}$$

By using (21) in [24], Equation (19) can be finally written as (16). □

**Remark** **2.** *The lower bound of SOP $P_{\mathrm{SOP}}^{L}$ is a monotonously increasing function with respect to the SNR threshold $\gamma_{\mathrm{th}}$. Therefore, the minimum and maximum values of the lower bound of SOP are given by $P_{\mathrm{SOP}}^{L,\min}=P_{\mathrm{SOP}}^{L}\left(\gamma_{\mathrm{th}}=0\right)$ and $P_{\mathrm{SOP}}^{L,\max}=P_{\mathrm{SOP}}^{L}\left(\gamma_{\mathrm{th}}\to \infty \right)=1$, respectively.*

**Remark** **3.** *The lower bound of the SOP, $P_{\mathrm{SOP}}^{L}$, is independent of the average transmit power P. Therefore, increasing P can not improve the performance of $P_{\mathrm{SOP}}^{L}$.*

### 3.2. Probability of SPSC Analysis

In physical layer secure communications, the probability of SPSC is often used as a fundamental benchmark, which is used to emphasize the probability of the existence of the secrecy capacity. In this paper, the probability of SPSC is given by setting $\gamma_{\mathrm{th}}=0$ in (12). Therefore, the following theorem is obtained.

**Theorem** **3.** *For the M-distributed fading channels (i.e., $H_{n}∼M\left(\alpha_{n},\beta_{n},\rho_{n},\Omega_{n},\xi_{n},\phi_{1,n},\phi_{2,n}\right)$, the closed-form expression of the probability of SPSC is given by*

(20) $$\begin{array}{c} P_{\mathrm{SPSC}}=1-\frac{A_{B}A_{E}}{4}\sum_{k=1}^{\beta_{E}}\sum_{j=1}^{\beta_{B}}a_{k,E}a_{j,B}{\left(\frac{\sigma_{B}}{\sigma_{E}}\right)}^{\frac{\alpha_{B}+j}{2}}{\left(\frac{\alpha_{E}\beta_{E}}{\xi_{g,E}\beta_{E}+\Omega_{E}^{\prime }}\right)}^{-\frac{\alpha_{E}+\alpha_{B}+k+j}{2}}G_{3,3}^{3,2}\left[\chi \left(0\right)\left|\begin{array}{c} 1-\frac{\alpha_{B}+j}{2}-\alpha_{E},1-\frac{\alpha_{B}+j}{2}-k,1-\frac{\alpha_{B}+j}{2}\\ \frac{\alpha_{B}-j}{2},-\frac{\alpha_{B}-j}{2},-\frac{\alpha_{B}+j}{2} \end{array}\right.\right] \end{array}$$

*where χ(·) is given by (17).*

**Proof.**  According to the definition of the probability of SPSC and (10), we have

(21) $$\begin{array}{ccc} P_{\mathrm{SPSC}} & = & P_{\mathrm{SOP}}\left(\gamma =0\right)\\ & = & \mathrm{Pr}\left[\gamma_{B}\leq \gamma_{E}\right] \end{array}$$

In (15), when γ=0, the lower bound of SOP is given by

(22) $$\begin{array}{c} P_{\mathrm{SOP}}^{L}\left(\gamma =0\right)=\mathrm{Pr}\left[\gamma_{B}\leq \gamma_{E}\right] \end{array}$$

Equations (21) and (22) imply that $P_{\mathrm{SPSC}}=P_{\mathrm{SOP}}^{L}\left(\gamma =0\right)$. Letting γ=0 in (16), (20) can be derived. □

**Remark** **4.** *Similar to* Remark 3*, the probability of SPSC, $P_{\mathrm{SPSC}}$, is also independent of the average transmit power P. This indicates that the probability of SPSC can not be improved by increasing P.*

### 3.3. ESC Analysis

Given the instantaneous SNRs at Bob and Eve, the instantaneous secrecy capacity is given by

(23) $$\begin{array}{c} C\left(u,w\right)={\left\{\ln(1+u)-\ln(1+w)\right\}}^{+} \end{array}$$

Then, the ESC can be written as

(24) $$\begin{array}{c} C_{\mathrm{Erg}}=\int_{0}^{\infty }\int_{0}^{\infty }C\left(u,v\right)f_{\gamma_{B}}\left(u\right)f_{\gamma_{E}}\left(w\right)dwdu \end{array}$$

By calculating (24), the following theorem is derived.

**Theorem** **4.** *For the M-distributed fading channels (i.e., $H_{n}∼M\left(\alpha_{n},\beta_{n},\rho_{n},\Omega_{n},\xi_{n},\phi_{1,n},\phi_{2,n}\right)$, the ESC is given by*

(25) $$\begin{array}{ccc} C_{\mathrm{Erg}} & = & \frac{A_{B}A_{E}}{4}\sum_{j=1}^{\beta_{B}}\sum_{k=1}^{\beta_{E}}a_{k,E}{\left(\frac{\sigma_{E}}{\sqrt{P}}\right)}^{\frac{\alpha_{E}+k}{2}}a_{j,B}{\left(\frac{\sigma_{B}}{\sqrt{P}}\right)}^{\frac{\alpha_{B}+j}{2}}\\ & \times & \int_{0}^{\infty }\ln\left(1+u\right)u^{\frac{\alpha_{B}+j+\alpha_{E}+k}{4}-1}K_{\alpha_{B}-j}\left(2\sqrt{\frac{\alpha_{B}\beta_{B}\sigma_{B}\sqrt{u}}{\sqrt{P}\left(\xi_{g,B}\beta_{B}+\Omega_{B}^{\prime }\right)}}\right)G_{1,3}^{2,1}\left[\frac{\alpha_{E}\beta_{E}\sigma_{E}\sqrt{u}}{\sqrt{P}\left(\xi_{g,E}\beta_{E}+\Omega_{E}^{\prime }\right)}\left|\begin{array}{c} 1-\frac{\alpha_{E}+k}{2}\\ \frac{\alpha_{E}-k}{2},\frac{k-\alpha_{E}}{2},-\frac{\alpha_{E}+k}{2} \end{array}\right.\right]du\\ & + & \frac{A_{E}A_{B}}{4}\sum_{k=1}^{\beta_{E}}\sum_{j=1}^{\beta_{B}}a_{k,E}{\left(\frac{\sigma_{E}}{\sqrt{P}}\right)}^{\frac{\alpha_{E}+k}{2}}a_{j,B}{\left(\frac{\sigma_{B}}{\sqrt{P}}\right)}^{\frac{\alpha_{B}+j}{2}}\\ & \times & \int_{0}^{\infty }\ln\left(1+w\right)w^{\frac{\alpha_{E}+k+\alpha_{B}+j}{4}-1}K_{\alpha_{E}-k}\left(2\sqrt{\frac{\alpha_{E}\beta_{E}\sigma_{E}\sqrt{w}}{\sqrt{P}\left(\xi_{g,E}\beta_{E}+\Omega_{E}^{\prime }\right)}}\right)G_{1,3}^{2,1}\left[\frac{\alpha_{B}\beta_{B}\sigma_{B}\sqrt{w}}{\sqrt{P}\left(\xi_{g,B}\beta_{B}+\Omega_{B}^{\prime }\right)}\left|\begin{array}{c} 1-\frac{\alpha_{B}+j}{2}\\ \frac{\alpha_{B}-j}{2},\frac{j-\alpha_{B}}{2},-\frac{\alpha_{B}+j}{2} \end{array}\right.\right]dw\\ & + & \frac{A_{E}}{8\pi }\sum_{k=1}^{\beta_{E}}a_{k,E}{\left(\frac{\sigma_{E}}{\sqrt{P}}\right)}^{\frac{\alpha_{E}+k}{2}}G_{2,6}^{6,1}\left[\frac{{\left(\alpha_{E}\beta_{E}\sigma_{E}\right)}^{2}}{16P{\left(\xi_{g,E}\beta_{E}+\Omega_{E}^{\prime }\right)}^{2}}\left|\begin{array}{c} -\frac{\alpha_{E}+k}{4},1-\frac{\alpha_{E}+k}{4}\\ \frac{\alpha_{E}-k}{4},\frac{\alpha_{E}-k+2}{4},\frac{k-\alpha_{E}}{4},\frac{2-\alpha_{E}+k}{4},-\frac{\alpha_{E}+k}{4},-\frac{\alpha_{E}+k}{4} \end{array}\right.\right] \end{array}$$

**Proof.**  According to (23) and (24), the ESC can be further expressed as

(26) $$\begin{array}{ccc} C_{\mathrm{Erg}} & = & \int_{0}^{\infty }\int_{0}^{u}\left[\ln(1+u)-\ln(1+w)\right]f_{\gamma_{B}}\left(u\right)f_{\gamma_{E}}\left(w\right)dwdu\\ & = & \underset{C_{1}}{\underset{︸}{\int_{0}^{\infty }\ln\left(1+u\right)f_{\gamma_{B}}\left(u\right)F_{\gamma_{E}}\left(u\right)du}}+\underset{C_{2}}{\underset{︸}{\int_{0}^{\infty }\ln\left(1+w\right)f_{\gamma_{E}}\left(w\right)F_{\gamma_{B}}\left(w\right)dw}}-\underset{C_{3}}{\underset{︸}{\int_{0}^{\infty }\ln\left(1+w\right)f_{\gamma_{E}}\left(w\right)dw}} \end{array}$$

where the first term $C_{1}$ can be written as

(27) $$\begin{array}{ccc} C_{1} & = & \frac{A_{B}A_{E}}{4}\sum_{j=1}^{\beta_{B}}\sum_{k=1}^{\beta_{E}}a_{k,E}{\left(\frac{\sigma_{E}}{\sqrt{P}}\right)}^{\frac{\alpha_{E}+k}{2}}a_{j,B}{\left(\frac{\sigma_{B}}{\sqrt{P}}\right)}^{\frac{\alpha_{B}+j}{2}}\int_{0}^{\infty }\ln\left(1+u\right)u^{\frac{\alpha_{B}+j+\alpha_{E}+k}{4}-1}K_{\alpha_{B}-j}\left(2\sqrt{\frac{\alpha_{B}\beta_{B}\sigma_{B}\sqrt{u}}{\sqrt{P}\left(\xi_{g,B}\beta_{B}+\Omega_{B}^{\prime }\right)}}\right)\\ & \times & G_{1.3}^{2,1}\left[\frac{\alpha_{E}\beta_{E}\sigma_{E}\sqrt{u}}{\sqrt{P}\left(\xi_{g,E}\beta_{E}+\Omega_{E}^{\prime }\right)}\left|\begin{array}{c} 1-\frac{\alpha_{E}+k}{2}\\ \frac{\alpha_{E}-k}{2},\frac{k-\alpha_{E}}{2},-\frac{\alpha_{E}+k}{2} \end{array}\right.\right]du \end{array}$$

Similarly, the second term $C_{2}$ can be expressed as

(28) $$\begin{array}{ccc} C_{2} & = & \frac{A_{E}A_{B}}{4}\sum_{k=1}^{\beta_{E}}\sum_{j=1}^{\beta_{B}}a_{k,E}{\left(\frac{\sigma_{E}}{\sqrt{P}}\right)}^{\frac{\alpha_{E}+k}{2}}a_{j,B}{\left(\frac{\sigma_{B}}{\sqrt{P}}\right)}^{\frac{\alpha_{B}+j}{2}}\int_{0}^{\infty }\ln\left(1+w\right)w^{\frac{\alpha_{E}+k+\alpha_{B}+j}{4}-1}K_{\alpha_{E}-k}\left(2\sqrt{\frac{\alpha_{E}\beta_{E}\sigma_{E}\sqrt{w}}{\sqrt{P}\left(\xi_{g,E}\beta_{E}+\Omega_{E}^{\prime }\right)}}\right)\\ & \times & G_{1.3}^{2,1}\left[\frac{\alpha_{B}\beta_{B}\sigma_{B}\sqrt{w}}{\sqrt{P}\left(\xi_{g,B}\beta_{B}+\Omega_{B}^{\prime }\right)}\left|\begin{array}{c} 1-\frac{\alpha_{B}+j}{2}\\ \frac{\alpha_{B}-j}{2},\frac{j-\alpha_{B}}{2},-\frac{\alpha_{B}+j}{2} \end{array}\right.\right]dw \end{array}$$

Moreover, the third term $C_{3}$ can be written as

(29) $$\begin{array}{c} C_{3}=\frac{A_{E}}{4}\sum_{k=1}^{\beta_{E}}a_{k,E}{\left(\frac{\sigma_{E}}{\sqrt{P}}\right)}^{\frac{\alpha_{E}+k}{2}}\int_{0}^{\infty }\ln(1+w)w^{\frac{\alpha_{E}+k}{4}-1}G_{0,2}^{2,0}\left[\frac{\alpha_{E}\beta_{E}\sigma_{E}\sqrt{w}}{\sqrt{P}\left(\xi_{g,E}\beta_{E}+\Omega_{E}^{\prime }\right)}\left|\begin{array}{c} -\\ \frac{\alpha_{E}-k}{2},-\frac{\alpha_{E}-k}{2} \end{array}\right.\right]dw \end{array}$$

According to (11) in [24], Equation (29) is further written as

(30) $$\begin{array}{ccc} C_{3} & = & \frac{A_{E}}{4}\sum_{k=1}^{\beta_{E}}a_{k,E}{\left(\frac{\sigma_{E}}{\sqrt{P}}\right)}^{\frac{\alpha_{E}+k}{2}}\int_{0}^{\infty }w^{\frac{\alpha_{E}+k}{4}-1}G_{2,2}^{1,2}\left[w\left|\begin{array}{c} 1,\\ 1, \end{array}\begin{array}{c} 1\\ 0 \end{array}\right.\right]G_{0,2}^{2,0}\left[\frac{\alpha_{E}\beta_{E}\sigma_{E}\sqrt{w}}{\sqrt{P}\left(\xi_{g,E}\beta_{E}+\Omega_{E}^{\prime }\right)}\left|\begin{array}{c} -\\ \frac{\alpha_{E}-k}{2},-\frac{\alpha_{E}-k}{2} \end{array}\right.\right]dw \end{array}$$

By employing (21) in [24], (30) can be further written as

(31) $$\begin{array}{c} C_{3}=\frac{A_{E}}{8\pi }\sum_{k=1}^{\beta_{E}}a_{k,E}{\left(\frac{\sigma_{E}}{\sqrt{P}}\right)}^{\frac{\alpha_{E}+k}{2}}G_{2,6}^{6,1}\left[\frac{{\left(\alpha_{E}\beta_{E}\sigma_{E}\right)}^{2}}{16P{\left(\xi_{g,E}\beta_{E}+\Omega_{E}^{\prime }\right)}^{2}}\left|\begin{array}{c} -\frac{\alpha_{E}+k}{4},1-\frac{\alpha_{E}+k}{4}\\ \frac{\alpha_{E}-k}{4},\frac{\alpha_{E}-k+2}{4},\frac{k-\alpha_{E}}{4},\frac{2-\alpha_{E}+k}{4},-\frac{\alpha_{E}+k}{4},-\frac{\alpha_{E}+k}{4} \end{array}\right.\right] \end{array}$$

Then, substituting (27), (28) and (31), (25) can be derived. □

It can be seen from *Theorem 4* that the exact expression of the ESC (25) is a very complex integral expression. It is very hard to obtain a closed-form expression for the ESC. Alternatively, a lower bound of the ESC is derived in the following theorem.

**Theorem** **5.** *For the M-distributed fading channels (i.e., $H_{n}∼M\left(\alpha_{n},\beta_{n},\rho_{n},\Omega_{n},\xi_{n},\phi_{1,n},\phi_{2,n}\right)$, a closed-form expression for the lower bound of the ESC is given by*

(32) $$\begin{array}{ccc} C_{\mathrm{Erg}}^{L} & = & \left\{\frac{A_{B}}{8\pi }\sum_{j=1}^{\beta_{B}}a_{j,B}{\left(\frac{\sigma_{B}}{\sqrt{P}}\right)}^{\frac{\alpha_{B}+j}{2}}\right.G_{2,6}^{6,1}\left[\frac{{\left(\alpha_{B}\beta_{B}\sigma_{B}\right)}^{2}}{16P{\left(\xi_{g,B}\beta_{B}+\Omega_{B}^{\prime }\right)}^{2}}\left|\begin{array}{c} -\frac{\alpha_{B}+j}{4},1-\frac{\alpha_{B}+j}{4}\\ \frac{\alpha_{B}-j}{4},\frac{\alpha_{B}-j+2}{4},\frac{j-\alpha_{B}}{4},\frac{2-\alpha_{B}+j}{4},-\frac{\alpha_{B}+j}{4},-\frac{\alpha_{B}+j}{4} \end{array}\right.\right]\\ & - & \frac{A_{E}}{8\pi }\sum_{k=1}^{\beta_{E}}a_{k,E}{\left(\frac{\sigma_{E}}{\sqrt{P}}\right)}^{\frac{\alpha_{E}+k}{2}}{\left.G_{2,6}^{6,1}\left[\frac{{\left(\alpha_{E}\beta_{E}\sigma_{E}\right)}^{2}}{16P{\left(\xi_{g,E}\beta_{E}+\Omega_{E}^{\prime }\right)}^{2}}\left|\begin{array}{c} -\frac{\alpha_{E}+k}{4},1-\frac{\alpha_{E}+k}{4}\\ \frac{\alpha_{E}-k}{4},\frac{\alpha_{E}-k+2}{4},\frac{k-\alpha_{E}}{4},\frac{2-\alpha_{E}+k}{4},-\frac{\alpha_{E}+k}{4},-\frac{\alpha_{E}+k}{4} \end{array}\right.\right]\right\}}^{+} \end{array}$$

**Proof.**  According to (31), the ergodic capacity for Alice-Bob link is given by

(33) $$\begin{array}{c} C_{1,\mathrm{Erg}}=\frac{A_{B}}{8\pi }\sum_{j=1}^{\beta_{B}}a_{j,B}{\left(\frac{\sigma_{B}}{\sqrt{P}}\right)}^{\frac{\alpha_{B}+j}{2}}G_{2,6}^{6,1}\left[\frac{{\left(\alpha_{B}\beta_{B}\sigma_{B}\right)}^{2}}{16P{\left(\xi_{g,B}\beta_{B}+\Omega_{B}^{\prime }\right)}^{2}}\left|\begin{array}{c} -\frac{\alpha_{B}+j}{4},1-\frac{\alpha_{B}+j}{4}\\ \frac{\alpha_{B}-j}{4},\frac{\alpha_{B}-j+2}{4},\frac{j-\alpha_{B}}{4},\frac{2-\alpha_{B}+j}{4},-\frac{\alpha_{B}+j}{4},-\frac{\alpha_{B}+j}{4} \end{array}\right.\right] \end{array}$$

Similarly, the ergodic capacity for Alice-Eve link is $C_{3}$ in (31).

Referring to [25], a lower bound of the ESC is given by

(34) $$\begin{array}{c} C_{\mathrm{Erg}}\geq {\left\{C_{1,\mathrm{Erg}}-C_{2,\mathrm{Erg}}\right\}}^{+} \end{array}$$

Substituting (33) and (31) into (34), (32) can be derived. □

## 4. Some Special Cases

As it is well known, the M distribution covers many well-known fading models as special cases, which is shown in Table 1. In this section, the derived secrecy analysis results in Section 3 will be extended to these fading channels.

### 4.1. K Distribution Channel

Letting $\Omega_{n}=0$ and $\rho_{n}=0$, the M distribution in (2) reduces to the K distribution as

(35) $$\begin{array}{c} f_{H_{n}}\left(h\right)=\frac{2\alpha_{n}^{\frac{\alpha_{n}+1}{2}}h^{\frac{\alpha_{n}-1}{2}}}{\Gamma \left(\alpha_{n}\right){\left(\xi_{g,n}\right)}^{\frac{\alpha_{n}+1}{2}}}K_{\alpha_{n}-1}\left(2\sqrt{\frac{h\alpha_{n}}{\xi_{g,n}}}\right),\;h\geq 0 \end{array}$$

In this case, the lower bound of SOP in (16) becomes

(36) $$\begin{array}{c} P_{\mathrm{SOP}}^{L}=1-\frac{{\left(1+\gamma_{\mathrm{th}}\right)}^{\frac{\alpha_{B}+1}{4}}}{\Gamma \left(\alpha_{E}\right)\Gamma \left(\alpha_{B}\right)}{\left(\frac{\sigma_{B}\alpha_{B}\xi_{g,E}}{\sigma_{E}\alpha_{E}\xi_{g,B}}\right)}^{\frac{\alpha_{B}+1}{2}}G_{3,3}^{3,2}\left[\frac{\sigma_{B}\alpha_{B}\xi_{g,E}\sqrt{1+\gamma_{\mathrm{th}}}}{\sigma_{E}\alpha_{E}\xi_{g,B}}\left|\begin{array}{c} \frac{1-2\alpha_{E}-\alpha_{B}}{2},-\frac{\alpha_{B}+1}{2},\frac{1-\alpha_{B}}{2}\\ \frac{\alpha_{B}-1}{2},\frac{1-\alpha_{B}}{2},-\frac{\alpha_{B}+1}{2} \end{array}\right.\right] \end{array}$$

Moreover, the probability of SPSC in (20) reduces to

(37) $$\begin{array}{c} P_{\mathrm{SPSC}}=1-\frac{1}{\Gamma \left(\alpha_{E}\right)\Gamma \left(\alpha_{B}\right)}{\left(\frac{\sigma_{B}\alpha_{B}\xi_{g,E}}{\sigma_{E}\alpha_{E}\xi_{g,B}}\right)}^{\frac{\alpha_{B}+1}{2}}G_{3,3}^{3,2}\left[\frac{\sigma_{B}\alpha_{B}\xi_{g,E}}{\sigma_{E}\alpha_{E}\xi_{g,B}}\left|\begin{array}{c} \frac{1-2\alpha_{E}-\alpha_{B}}{2},-\frac{\alpha_{B}+1}{2},\frac{1-\alpha_{B}}{2}\\ \frac{\alpha_{B}-1}{2},\frac{1-\alpha_{B}}{2},-\frac{\alpha_{B}+1}{2} \end{array}\right.\right] \end{array}$$

Furthermore, the lower bound of ESC in (32) becomes

(38) $$\begin{array}{c} C_{\mathrm{Erg}}^{L}=\left\{{\left(\frac{\alpha_{B}\sigma_{B}}{\xi_{g,B}\sqrt{P}}\right)}^{\frac{\alpha_{B}+1}{2}}\frac{1}{4\pi \Gamma (\alpha_{B})}\right.G_{2,6}^{6,1}\left[\left.\frac{1}{16}{\left(\frac{\alpha_{B}\sigma_{B}}{\xi_{g,B}\sqrt{P}}\right)}^{2}\right|\begin{array}{c} -\frac{\alpha_{B}+1}{4},\frac{3-\alpha_{B}}{4}\\ \frac{\alpha_{B}-1}{4},\frac{\alpha_{B}+1}{4},\frac{1-\alpha_{B}}{4},\frac{3-\alpha_{B}}{4},-\frac{\alpha_{B}+1}{4},-\frac{\alpha_{B}+1}{4} \end{array}\right]\\ -{\left(\frac{\alpha_{E}\sigma_{E}}{\xi_{g,E}\sqrt{P}}\right)}^{\frac{\alpha_{E}+1}{2}}\frac{1}{4\pi \Gamma (\alpha_{E})}{\left.G_{2,6}^{6,1}\left[\left.\frac{1}{16}{\left(\frac{\alpha_{E}\sigma_{E}}{\xi_{g,E}\sqrt{P}}\right)}^{2}\right|\begin{array}{c} -\frac{\alpha_{E}+1}{4},\frac{3-\alpha_{E}}{4}\\ \frac{\alpha_{E}-1}{4},\frac{\alpha_{E}+1}{4},\frac{1-\alpha_{E}}{4},\frac{3-\alpha_{E}}{4},-\frac{\alpha_{E}+1}{4},-\frac{\alpha_{E}+1}{4} \end{array}\right]\right\}}^{+} \end{array}$$

### 4.2. Exponential Distribution Channel

When Ω=0, ρ=0 and α→∞, the M distribution in (2) reduces to the exponential distribution

(39) $$\begin{array}{c} f_{H,n}\left(h\right)=\frac{1}{\xi_{g,n}}\exp\left(-\frac{h}{\xi_{g,n}}\right),\;h\geq 0 \end{array}$$

Under this channel, the lower bound of SOP in (16) becomes

(40) $$\begin{array}{c} P_{\mathrm{SOP}}^{L}=\frac{\xi_{g,E}\sigma_{B}\sqrt{1+\gamma_{\mathrm{th}}}}{\xi_{g,B}\sigma_{E}+\xi_{g,E}\sigma_{B}\sqrt{1+\gamma_{\mathrm{th}}}} \end{array}$$

Moreover, the probability of SPSC in (20) reduces to

(41) $$\begin{array}{c} P_{\mathrm{SPSC}}=\frac{\xi_{g,E}\sigma_{B}}{\xi_{g,B}\sigma_{E}+\xi_{g,E}\sigma_{B}} \end{array}$$

Furthermore, the lower bound of ESC in (32) becomes

(42) $$\begin{array}{c} C_{\mathrm{Erg}}^{L}=\left\{\frac{\sigma_{B}}{2\xi_{g,B}\sqrt{P\pi }}G_{2,4}^{4,1}\left[\left.{\left(\frac{\sigma_{B}}{2\xi_{g,B}\sqrt{P}}\right)}^{2}\right|\begin{array}{c} -\frac{1}{2},\frac{1}{2}\\ 0,\frac{1}{2},-\frac{1}{2},-\frac{1}{2} \end{array}\right]\right.-{\left.\frac{\sigma_{E}}{2\xi_{g,E}\sqrt{\pi P}}G_{2,4}^{4,1}\left[\left.{\left(\frac{\sigma_{E}}{2\xi_{g,E}\sqrt{P}}\right)}^{2}\right|\begin{array}{c} -\frac{1}{2},\frac{1}{2}\\ 0,\frac{1}{2},-\frac{1}{2},-\frac{1}{2} \end{array}\right]\right\}}^{+} \end{array}$$

### 4.3. Gamma-Gamma Distribution Channels

The Gamma-Gamma distribution is derived by letting ρ=1 and $\Omega^{\prime }=1$ in the M distribution (2). Therefore, the PDF of the Gamma-Gamma distribution is written as

(43) $$\begin{array}{c} f_{H_{n}}\left(h\right)=\frac{2{\left(\alpha_{n}\beta_{n}\right)}^{\frac{\alpha_{n}+\beta_{n}}{2}}}{\Gamma \left(\alpha_{n}\right)\Gamma \left(\beta_{n}\right)}h^{\frac{\alpha_{n}+\beta_{n}}{2}-1}K_{\alpha_{n}-\beta_{n}}\left(2\sqrt{\alpha_{n}\beta_{n}h}\right),\;h\geq 0 \end{array}$$

With this channel, the lower bound of SOP in (16) becomes

(44) $$\begin{array}{c} P_{\mathrm{SOP}}^{L}=1-\frac{{\left(1+\gamma_{\mathrm{th}}\right)}^{\frac{\alpha_{B}+\beta_{B}}{4}}}{\Gamma \left(\alpha_{B}\right)\Gamma \left(\beta_{B}\right)\Gamma \left(\alpha_{E}\right)\Gamma \left(\beta_{E}\right)}{\left(\frac{\alpha_{B}\beta_{B}\sigma_{B}}{\alpha_{E}\beta_{E}\sigma_{E}}\right)}^{\frac{\alpha_{B}+\beta_{B}}{2}}G_{3,3}^{3,2}\left[\left.\frac{\alpha_{B}\beta_{B}\sigma_{B}\sqrt{1+\gamma_{\mathrm{th}}}}{\alpha_{E}\beta_{E}\sigma_{E}}\right|\begin{array}{c} \frac{2-2\alpha_{E}-\alpha_{B}-\beta_{B}}{2},\frac{2-2\beta_{E}-\alpha_{B}-\beta_{B}}{2},\frac{2-\alpha_{B}-\beta_{B}}{2}\\ \frac{\alpha_{B}-\beta_{B}}{2},\frac{\beta_{B}-\alpha_{B}}{2},-\frac{\alpha_{B}+\beta_{B}}{2} \end{array}\right] \end{array}$$

Moreover, the probability of SPSC in (20) reduces to

(45) $$\begin{array}{c} P_{\mathrm{PSPC}}=1-\frac{1}{\Gamma \left(\alpha_{B}\right)\Gamma \left(\beta_{B}\right)\Gamma \left(\alpha_{E}\right)\Gamma \left(\beta_{E}\right)}{\left(\frac{\alpha_{B}\beta_{B}\sigma_{B}}{\alpha_{E}\beta_{E}\sigma_{E}}\right)}^{\frac{\alpha_{B}+\beta_{B}}{2}}G_{3,3}^{3,2}\left[\left.\frac{\alpha_{B}\beta_{B}\sigma_{B}}{\alpha_{E}\beta_{E}\sigma_{E}}\right|\begin{array}{c} \frac{2-2\alpha_{E}-\alpha_{B}-\beta_{B}}{2},\frac{2-2\beta_{E}-\alpha_{B}-\beta_{B}}{2},\frac{2-\alpha_{B}-\beta_{B}}{2}\\ \frac{\alpha_{B}-\beta_{B}}{2},\frac{\beta_{B}-\alpha_{B}}{2},-\frac{\alpha_{B}+\beta_{B}}{2} \end{array}\right] \end{array}$$

Furthermore, the lower bound of ESC in (32) becomes

(46) $$\begin{array}{ccc} C_{\mathrm{Erg}}^{L} & = & \left\{\frac{1}{4\pi \Gamma \left(\alpha_{B}\right)\Gamma \left(\beta_{B}\right)}{\left(\frac{\alpha_{B}\beta_{B}\sigma_{B}}{\sqrt{P}}\right)}^{\frac{\alpha_{B}+\beta_{B}}{2}}G_{2,6}^{6,1}\left[\left.{\frac{\left(\alpha_{B}\beta_{B}\sigma_{B}\right)}{16P}}^{2}\right|\begin{array}{c} -\frac{\alpha_{B}+\beta_{B}}{4},1-\frac{\alpha_{B}+\beta_{B}}{4}\\ \frac{\alpha_{B}-\beta_{B}}{4},\frac{\alpha_{B}-\beta_{B}+2}{4},\frac{\beta_{B}-\alpha_{B}}{4},\frac{\beta_{B}-\alpha_{B}+2}{4},-\frac{\alpha_{B}+\beta_{B}}{4},-\frac{\alpha_{B}+\beta_{B}}{4} \end{array}\right]\right.\\ & - & \frac{1}{4\pi \Gamma \left(\alpha_{E}\right)\Gamma \left(\beta_{E}\right)}{\left(\frac{\alpha_{E}\beta_{E}\sigma_{E}}{\sqrt{P}}\right)}^{\frac{\alpha_{E}+\beta_{E}}{2}}{\left.G_{2,6}^{6,1}\left[\left.{\frac{\left(\alpha_{E}\beta_{E}\sigma_{E}\right)}{16P}}^{2}\right|\begin{array}{c} -\frac{\alpha_{E}+\beta_{E}}{4},1-\frac{\alpha_{E}+\beta_{E}}{4}\\ \frac{\alpha_{E}-\beta_{E}}{4},\frac{\alpha_{E}-\beta_{E}+2}{4},\frac{\beta_{E}-\alpha_{E}}{4},\frac{\beta_{E}-\alpha_{E}+2}{4},-\frac{\alpha_{E}+\beta_{E}}{4},-\frac{\alpha_{E}+\beta_{E}}{4} \end{array}\right]\right\}}^{+} \end{array}$$

### 4.4. Other Channels

In a similar manner, the secrecy performance for other fading channels in Table 1 can also be obtained. Due to the space limitation, the detailed derivations are omitted here.

## 5. Numerical Results

In this section, a wireless communication system with Alice, Bob and Eve are considered. Under the system model, some typical results will be presented. To evaluate the secure performance of the system, the average strengths of Alice-Bob link and Alice-Eve link are used as the performance metrics. The two performance indicators are given as

(47) $$\begin{array}{c} \left\{\begin{array}{c} E\left(\gamma_{B}\right)=\frac{A_{B}P}{2\sigma_{B}^{2}}\Gamma \left(\alpha_{B}+2\right)\sum_{j=1}^{\beta_{B}}a_{B,j}{\left(\frac{\alpha_{B}\beta_{B}}{\xi_{g,B}\beta_{B}+\Omega_{B}^{\prime }}\right)}^{-\frac{\alpha_{B}+j+4}{2}}\Gamma \left(j+2\right)\\ E\left(\gamma_{E}\right)=\frac{A_{E}P}{2\sigma_{E}^{2}}\Gamma \left(\alpha_{E}+2\right)\sum_{k=1}^{\beta_{E}}a_{E,k}{\left(\frac{\alpha_{E}\beta_{E}}{\xi_{g,E}\beta_{E}+\Omega_{E}^{\prime }}\right)}^{-\frac{\alpha_{E}+k+4}{2}}\Gamma \left(k+2\right) \end{array}\right. \end{array}$$

In this section, the exact simulation results, the simulation results of the lower bound, and the theoretical results of the lower bound of different performance indicators will be shown. To facilitate the accuracy analysis, the relative error is employed as a useful measure. Here, three relative errors are defined as follows

(48) $$\begin{array}{c} \left\{\begin{array}{c} e_{1}=\frac{|S-T_{\mathrm{Low}}|}{S}\times 100\%\\ e_{2}=\frac{|S_{\mathrm{Low}}-T_{\mathrm{Low}}|}{S_{\mathrm{Low}}}\times 100\%\\ e_{3}=\frac{\left|S-T\right|}{S}\times 100\% \end{array}\right. \end{array}$$

where *S* and *T* denote the exact simulation value and theoretical value, $S_{\mathrm{Low}}$ and $T_{\mathrm{Low}}$ denote the simulation value and theoretical value of the lower bound.

### 5.1. SOP Results

**Figure 3:**
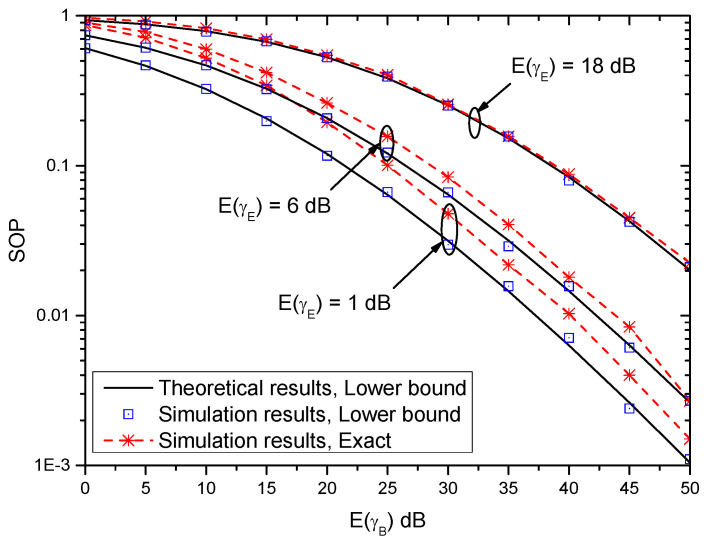
Secrecy outage probability (SOP) versus $E(\gamma_{B})$ with different $E(\gamma_{E})$ when $\gamma_{\mathrm{th}}=0$ dB and $\left(\alpha_{B},\beta_{B}\right)=\left(\alpha_{E},\beta_{E}\right)=\left(2,2\right)$.

Figure 3 shows the SOP versus $E(\gamma_{B})$ with different $E(\gamma_{E})$ when $\gamma_{\mathrm{th}}=0$ dB and $\left(\alpha_{B},\beta_{B}\right)=\left(\alpha_{E},\beta_{E}\right)=\left(2,2\right)$. It can be observed that the SOP performance improves with the increase of $E(\gamma_{B})$. This indicates that the larger the average strength of the main channel is, the better the system performance becomes. Moreover, the theoretical results of the lower bound of SOP match the simulation results very well. This shows the accuracy of the lower bound of SOP. Furthermore, when $E(\gamma_{E})=1$ dB, which does not satisfy the condition $E(\gamma_{E})\gg 1$, and thus the performance gap between the lower bound of SOP and the exact result of the SOP is observable. With the increase of $E(\gamma_{E})$, the gap becomes smaller and smaller. This indicates that when $E(\gamma_{E})\gg 1$, the derived lower bound of SOP can be used to evaluate the system performance, without reliable on time-intensive simulations.

**Figure 4:**
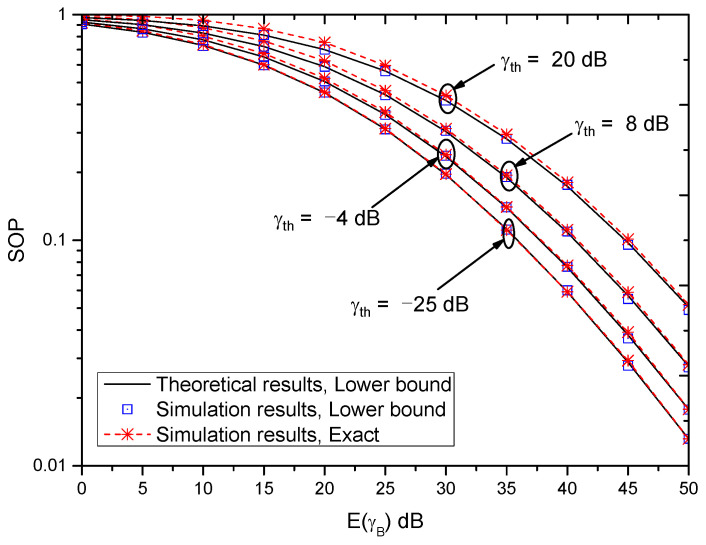
SOP versus $E(\gamma_{B})$ with different $\gamma_{\mathrm{th}}$ when $E(\gamma_{E})=18$ dB and $\left(\alpha_{B},\beta_{B}\right)=\left(\alpha_{E},\beta_{E}\right)=\left(2,2\right)$.

Figure 4 shows the SOP versus $E(\gamma_{B})$ with different $\gamma_{\mathrm{th}}$ when $E(\gamma_{W})=18$ dB and $\left(\alpha_{B},\beta_{B}\right)=\left(\alpha_{E},\beta_{E}\right)=\left(2,2\right)$. As can be observed, the SOP decreases with the increase of $E(\gamma_{B})$, which coincides with the conclusion in Figure 3. Moreover, for the lower bound of SOP, the gap between theoretical results and simulation results is very small, which verifies the accuracy of the derived lower bound. In this simulation, the lower bound of SOP and the exact result of the SOP almost coincide for larger $E(\gamma_{B})$ and $E(\gamma_{E})$, which shows the tightness of the lower bound of the SOP. Furthermore, it can be seen that the value of the SOP increases with the increase of $\gamma_{\mathrm{th}}$. This indicates that large SNR threshold will degrade the system performance.

Table 2 shows the accuracy of the SOP for different $E(\gamma_{B})$ when $E(\gamma_{E})=18$ dB, $\left(\alpha_{B},\beta_{B}\right)=\left(\alpha_{E},\beta_{E}\right)=\left(2,2\right)$ and $\gamma_{\mathrm{th}}=-4$ dB. As can be seen, all the relative errors $e_{1}$ and $e_{2}$ are quite small. This indicates that the derived lower bound of SOP in (16) is very accurate to evaluate the system performance.

**Figure 5:**
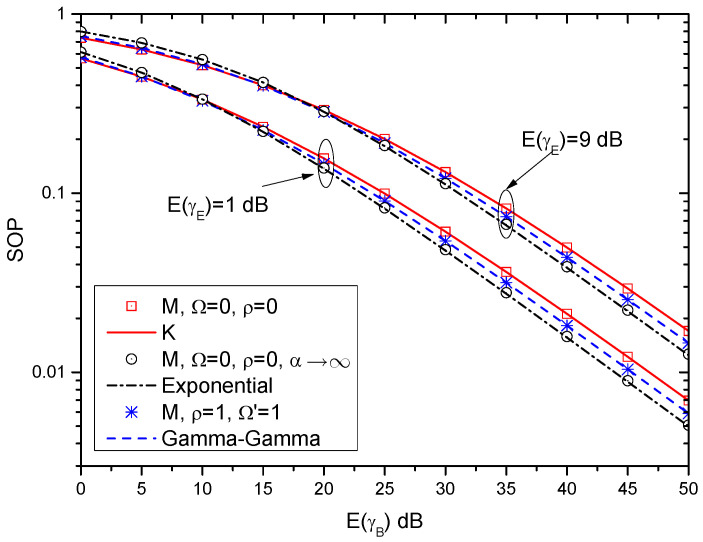
SOP versus $E(\gamma_{B})$ for different fading channels with $\gamma_{\mathrm{th}}=1$ dB.

In this subsection, the lower bounds of the SOP over different fading channels are also provided, as shown in Figure 5. In this figure, the lower bounds of the SOP over the K, exponential, and Gamma-Gamma fading channels are derived in (36), (40) and (44), respectively. Moreover, the lower bounds of the SOP over the M-distributed fading channel (i.e., (16)) with special parameters are also provided. As can be seen in Table 1, when Ω=0 and ρ=0, the M-distributed channel reduces to the K channel; when Ω=0, ρ=0 and α→∞, the M-distributed channel reduces to the exponential channel; when ρ=1 and $\Omega^{\prime }=1$, the M-distributed channel reduces to the Gamma-Gamma channel. As can be observed, the results of (16) with Ω=0 and ρ=0 match with that of (36) very well. The results of (16) with Ω=0, ρ=0 and α→∞ and the results of (40) coincide with each other. Similarly, the difference between (16) with ρ=1 and $\Omega^{\prime }=1$ and (44) is so small and it can be ignored. These conclusions indicate that the derived expression of SOP over the M-distributed fading channel is a general expression, which can be accurately extended to other well-known fading channels.

### 5.2. Probability of SPSC Results

**Figure 6:**
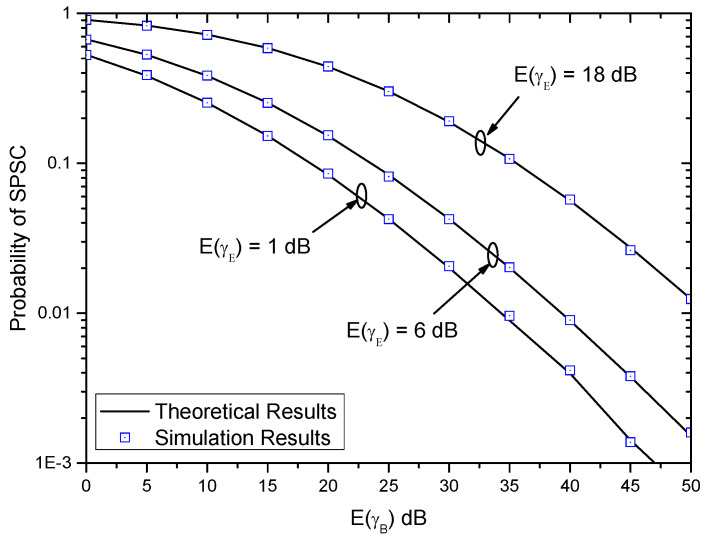
Probability of strictly positive secrecy capacity (SPSC) versus $E(\gamma_{B})$ with different $E(\gamma_{E})$ when $\left(\alpha_{B},\beta_{B}\right)=\left(\alpha_{E},\beta_{E}\right)=\left(2,2\right)$.

Figure 6 shows the probability of SPSC versus $E(\gamma_{B})$ with different $E(\gamma_{E})$ when $\left(\alpha_{B},\beta_{B}\right)=\left(\alpha_{E},\beta_{E}\right)=\left(2,2\right)$. In this figure, the value of SPSC decreases with the increase of $E(\gamma_{B})$. However, the value of the probability of SPSC increases with the increase of $E(\gamma_{E})$. This shows the impacts of the average strength of the main channel and the average strength of the main channel on system performance. Moreover, it can be seen that the theoretical results match the simulation results very well, and thus the derived exact expression of the probability of the SPSC is very accurate to evaluate the system performance.

**Figure 7:**
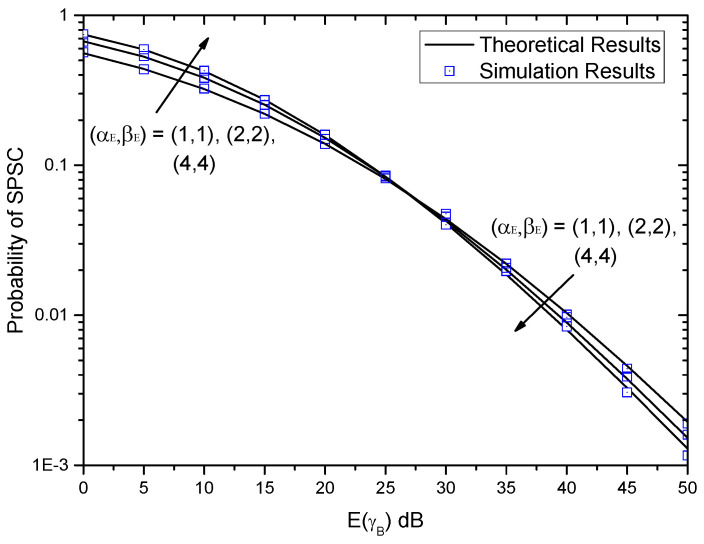
Probability of SPSC versus $E(\gamma_{B})$ with different $\left(\alpha_{E},\beta_{E}\right)$ when $\left(\alpha_{B},\beta_{B}\right)=\left(2,2\right)$.

Figure 7 shows the probability of SPSC versus $E(\gamma_{B})$ with different $\left(\alpha_{E},\beta_{E}\right)$ when $\left(\alpha_{B},\beta_{B}\right)=\left(2,2\right)$. Obviously, with the increase of $E(\gamma_{B})$, the probability of SPSC decreases. The conclusion is the same as that in Figure 6. Once again, it can be observed that small differences appear between the theoretical results and the simulation results. Therefore, the exact expression of the probability of SPSC can be used to evaluate the system performance without time-intensive simulations. Moreover, it can be seen that when $E(\gamma_{B})$ is small, the probability of SPSC increases with the increase of $\left(\alpha_{E},\beta_{E}\right)$. However, the trends of curves changes when $E(\gamma_{B})$ is large, i.e., the probability of SPSC decreases with the increase of $\left(\alpha_{E},\beta_{E}\right)$.

Table 3 shows the accuracy of the SOP for different $E(\gamma_{B})$ when $\left(\alpha_{B},\beta_{B}\right)=\left(2,2\right)$ and $\left(\alpha_{E},\beta_{E}\right)=\left(1,1\right)$. It can be seen that the discrepancies between the simulation and theoretical results are small enough in most cases. Therefore, the derived closed-form expression of the probability of SPSC is accuracy to evaluate the system performance.

**Figure 8:**
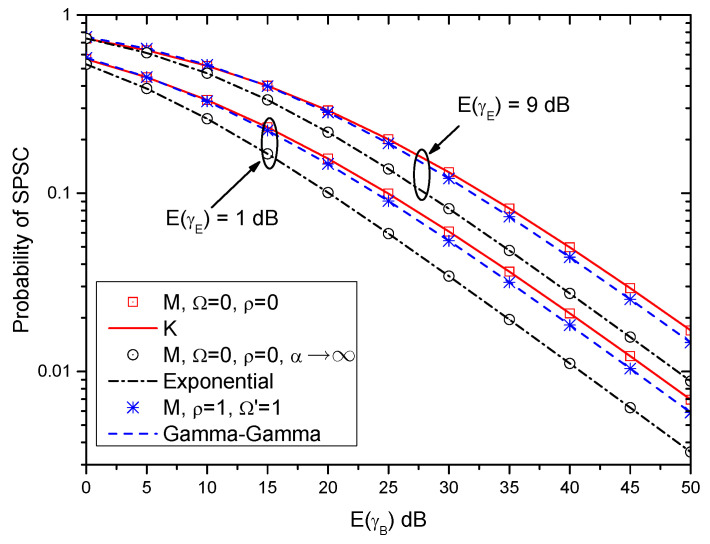
Probability of SPSC versus $E(\gamma_{B})$ for different fading channels.

In Figure 8, the probabilities of the SPSC over different fading channels are also provided. Specifically, the probabilities of the SPSC over the K, exponential, and Gamma-Gamma fading channels are obtained by (37), (41) and (45), respectively. Moreover, the probability of the SPSC over the M-distributed fading channel (i.e., (20)) with special parameters are also provided as shown in Table 1. Similar to Figure 5, the results over the M-distributed fading channel with special parameters match the results of the other fading channels very well. Therefore, the derived probability of SPSC over the M-distributed fading channel can be used as a unified expression for many well-known fading channels.

### 5.3. ESC Results

**Figure 9:**
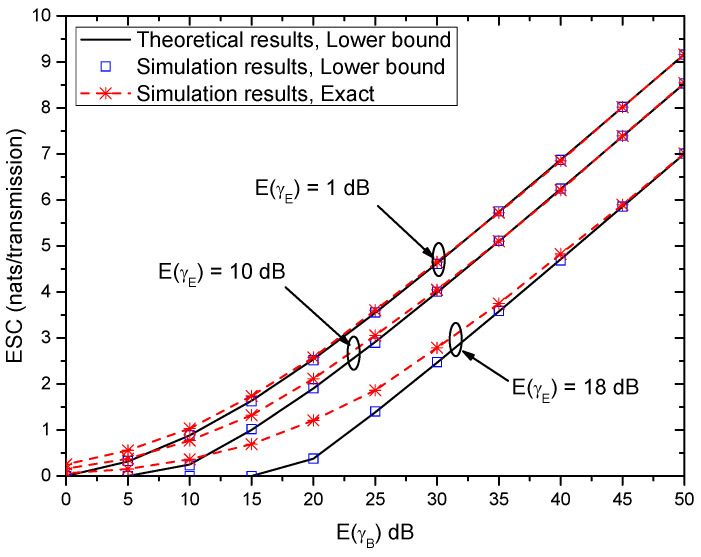
Ergodic secrecy capacity (ESC) versus $E(\gamma_{B})$ with different $E(\gamma_{E})$ when $\left(\alpha_{B},\beta_{B}\right)=\left(\alpha_{E},\beta_{E}\right)=\left(2,2\right)$.

Figure 9 shows the ESC versus $E(\gamma_{B})$ with different $E(\gamma_{E})$ when $\left(\alpha_{B},\beta_{B}\right)=\left(\alpha_{E},\beta_{E}\right)=\left(2,2\right)$. In Figure 9, when $E(\gamma_{B})$ is small, the ESC is almost zero. With the increase of $E(\gamma_{B})$, the ESC increases accordingly. For a fixed $E(\gamma_{B})$, the ESC performance degrades with the increase of $E(\gamma_{E})$. Moreover, for the lower bound of the ESC, the performance gap between theoretical results and simulation results is very mall, and thus the derived lower bound of ESC is accurate. Furthermore, when $E(\gamma_{B})$ is small, the difference between the lower bound of ESC and the exact results of ESC is large. However, with the increase of $E(\gamma_{B})$, the gap becomes smaller and smaller. Therefore, at high SNR regime, the derived lower bound of ESC can be employed to evaluate the system performance without time-intensive simulations.

**Figure 10:**
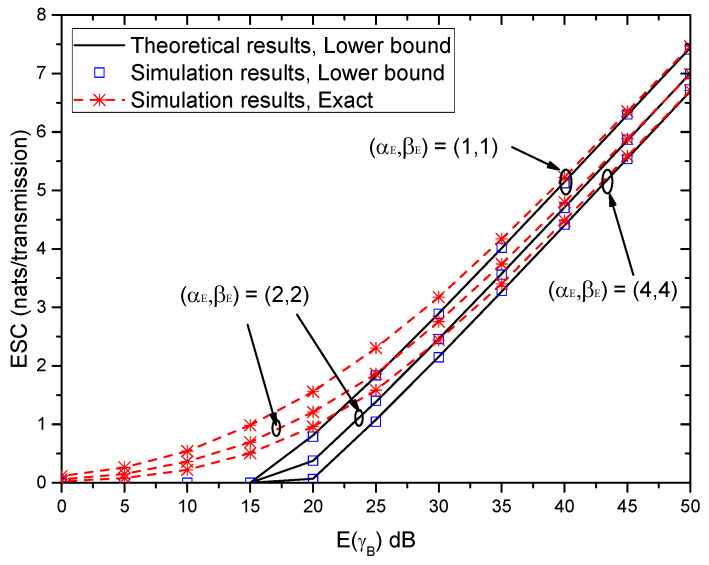
ESC versus $E(\gamma_{B})$ with different $\left(\alpha_{E},\beta_{E}\right)$ when $\left(\alpha_{B},\beta_{B}\right)=\left(2,2\right)$.

Figure 10 shows the ESC versus $E(\gamma_{B})$ with different $\left(\alpha_{E},\beta_{E}\right)$ when $\left(\alpha_{B},\beta_{B}\right)=\left(2,2\right)$. As seen in Figure 9, the ESC also increases with the increase of $E(\gamma_{B})$ in Figure 10. Moreover, the gaps among the theoretical results of the lower bound of ESC, the simulation results of the lower bound of ESC and the exact results of ESC are very small for large E(U). This conclusion is the same as that in Figure 9. Furthermore, with the increase of $\left(\alpha_{E},\beta_{E}\right)$, the ESC performance degrades.

Table 4 shows the relative errors of ESC when $\left(\alpha_{B},\beta_{B}\right)=\left(2,2\right)$ and $\left(\alpha_{E},\beta_{E}\right)=\left(4,4\right)$. It can be observed that the error $e_{2}$ is small for all cases. However, when $E(\gamma_{B})$ is small, the error $e_{1}$ is large. Small $E(\gamma_{B})$ represents the main channel is much worse than the eavesdropping channel, which is quite rare in practical scenarios. Moreover, with the increase of $E(\gamma_{B})$, $e_{1}$ decreases rapidly. Therefore, the derived lower bound of ESC is valid in the high SNR regime.

**Figure 11:**
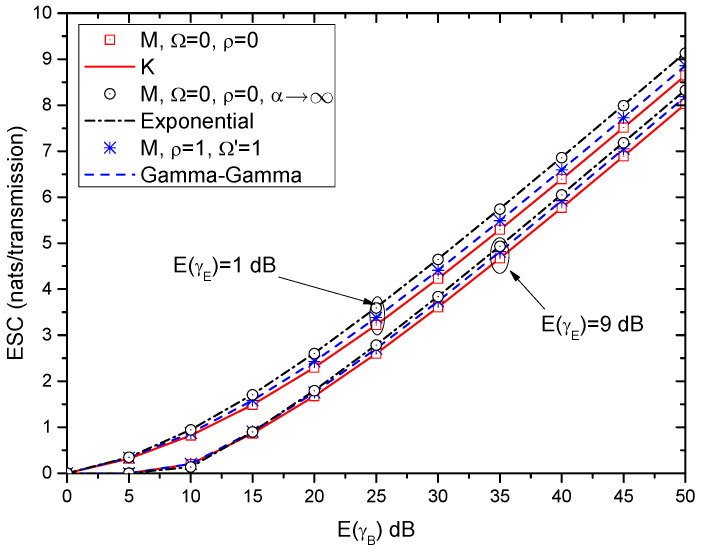
ESC versus $E(\gamma_{B})$ for different fading channels.

Furthermore, Figure 11 shows the lower bound of ESC versus $E(\gamma_{B})$ for different fading channels. The lower bounds of ESC over the K, exponential, and Gamma-Gamma fading channels are obtained by (38), (42) and (46), respectively. Moreover, the lower bound of ESC over the M-distributed fading channel (i.e., (25)) with special parameters are also provided as shown in Table 1. Once again, it can be seen that the results over the M-distributed fading channel with special parameters show close agreement with that of the other fading channels.

## 6. Conclusions

By considering the M-distributed fading channels, this paper has investigated the PLS problem. The theoretical expressions of the SOP, the probability of SPSC, and the ESC are derived, respectively. In this paper, a limitation of the theoretical analysis is that both the exact SOP and ESC are with a complex integral, and closed-form expressions for them are not derived. Alternatively, tight lower bounds for SOP and ESC are derived, which are in closed-forms. Numerical results show that the SOP and its lower bound are monotonously increasing functions with respect to $\gamma_{\mathrm{th}}$. Moreover, when $E(\gamma_{B})$ and $E(\gamma_{E})$ are large, the gap between the SOP and its lower bound is very small. The exact expression for probability of SPSC has the same form as that for the lower bound of SOP when $\gamma_{\mathrm{th}}=0$. With the increase of $\left(\alpha_{E},\beta_{E}\right)$, the probability of SPSC increases for small $E(\gamma_{B})$ and decreases for lagre $E(\gamma_{B})$. Moreover, for large $E(\gamma_{B})$ and $E(\gamma_{E})$, the difference between the exact ESC and its lower bound is small enough. Therefore, the derived expressions are accurate and can be used to evaluate the system performance without time-intensive simulations. Moreover, the derived results are extended to many well-known channels, which can provide some insights for practical system design.

After deriving the expressions of the secure performance indicators over the M-distributed fading channel, the natural next step is to consider the secure transmission schemes to further improve the PLS.

## Figures and Tables

**Table 1:** Special cases of the M distribution.

Distribution Models	Generation
K distribution	Ω=0, ρ=0
Exponential distribution	Ω=0, ρ=0, α→∞
Gamma-Gamma distribution	ρ=1, $\Omega^{\prime }=1$
Lognormal distribution	ρ=0, $\mathrm{Var}[|U_{L}|]=0$, $\xi_{g}\to 0$
Gamma-Rician distribution	β→∞
Gamma distribution	ρ=0, $\xi_{g}=0$
Rice-Nakagami distribution	ρ=0, $\mathrm{Var}[|U_{L}|]=0$

**Table 2:** Relative errors of SOP.

$E(\gamma_{B})$	10 dB	20 dB	30 dB	40 dB	50 dB
$e_{1}$	3.699%	3.205%	1.880%	1.936%	0.293%
$e_{2}$	0.436%	0.041%	0.795%	0.651%	0.156%

**Table 3:** Relative errors of the probability of SPSC.

$E(\gamma_{B})$	10 dB	20 dB	30 dB	40 dB	50 dB
$e_{3}$	0.780%	0.125%	7.777%	6.753%	2.283%

**Table 4:** Relative errors of ESC.

$E(\gamma_{B})$	25 dB	30 dB	35 dB	40 dB	45 dB	50 dB
$e_{1}$	31.911%	11.668%	3.879%	1.845%	0.713%	0.195%
$e_{2}$	3.112%	0.401%	0.419%	0.258%	0.221%	0.450%
